# Design of a Controlled Trial of Cascade Screening for Hypercholesterolemia: The (CASH) Study

**DOI:** 10.3390/jpm8030027

**Published:** 2018-08-23

**Authors:** Iftikhar J. Kullo, Kent R. Bailey

**Affiliations:** 1 Department of Cardiovascular Diseases and the Gonda Vascular Center, Mayo Clinic, Rochester, MN 55902, USA; 2 Department of Health Sciences Research (KRB), Mayo Clinic, Rochester, MN 55902, USA; baileyk@mayo.edu

**Keywords:** clinical trial, design, familial hypercholesterolemia, cascade screening, genetics, genetic risk, genetic testing, personalized medicine

## Abstract

To inform guidelines for screening family members of patients with familial hypercholesterolemia (FH), we designed a clinical trial to compare the yield of cascade screening in FH patients with and without an identifiable pathogenic variant. Participants with hypercholesterolemia (Low-density lipoprotein cholesterol (LDL-C) > 155 mg/dL) underwent sequencing of *LDLR*, *APOB*, and *PCSK9* and genotyping of six single nucleotide polymorphisms associated with LDL-C followed by calculation of a polygenic score for LDL-C. We identified 24 patients with definite FH (pathogenic variant in one of the three FH genes), 76 patients with probable FH (Dutch lipid clinic network (DLCN) score ≥ 6, no pathogenic variant), and 262 patients with possible FH (DLCN score 3–5, no pathogenic variant). We will enroll 50 patients with definite FH by recruiting an additional 26 from the FH Clinic at Mayo and 50 patients each with probable and possible FH, matching on age and sex. Family members of patients with definite FH will undergo testing for the relevant pathogenic variant using saliva kits and family members of those with probable/possible FH will have a lipid profile checked. We will assess the number of new cases detected (defined as presence of a pathogenic variant in the family member of definite FH patient or LDL-C > 155 mg/dL (>130 mg/dL in children) in family members of probable/possible FH patients, and the cost of detecting a new case. The proposed clinical trial will compare the yield and cost of cascade screening for FH patients with/without an identifiable pathogenic variant, and thereby inform guidelines for cascade screening for FH.

## 1. Introduction

Familial hypercholesterolemia (FH) is a relatively prevalent genetic disorder associated with increased risk of coronary heart disease (CHD) [1]. Early detection and treatment is needed to prevent adverse outcomes such as myocardial infarction and sudden cardiac death. The diagnosis of FH is often based on the Dutch lipid clinic network (DLCN) criteria [2] as follows: definite FH = score ≥ 8, probable = score ≥ 6, possible = score ≥ 3. Although FH often clusters in families, only a proportion (3–50%) has a mutation in *LDLR*, *APOB*, or *PCSK9* [3]. Current guidelines recommend cascade screening of family members of patients with FH [4]. However, the effectiveness of such screening in various categories of FH patients, for example those who do not have an identifiable pathogenic/likely pathogenic (P/LP) variant in an FH-related gene (FH^PV−^), is unclear. Little or no data are available on how healthcare providers should counsel probands with FH who do not have an identifiable P/LP variant regarding screening of family members. A proportion of such patients may have a yet unidentified monogenic etiology.

We hypothesized that although the number of new cases detected by cascade screening of families of patients with P/LP variants (FH^PV+^) will be less than for families of FH^PV−^ patients, such screening will have utility and will be cost-effective. To test this hypothesis, we designed a clinical trial to compare the yield of cascade screening and cost effectiveness in FH patients with or without a P/LP variant, the cascade screening in hypercholesterolemia (CASH) study. The proposed clinical trial, by providing hitherto unknown data regarding the yield and cost effectiveness of cascade screening for definite, probable or possible FH, a prevalent condition that is associated with significantly increased risk of CHD, will inform public health policy related to screening for FH.

## 2. Materials and Methods

The study was approved by the Mayo Clinic Institutional Review Board (IRB) in February 2017. The trial is registered at ClinicalTrials.gov (NCT03640234).

### 2.1. Overall Study Design

**Figure 1:**
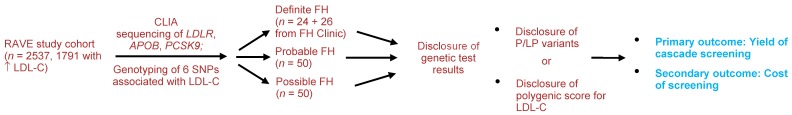
A flow diagram illustrating the design of the cascade screening in hypercholesterolemia (CASH) study. RAVE: return of actionable variants empiric study; LDL-C: low density lipoprotein cholesterol; CLIA: central laboratory improvement amendment; SNPs: single nucleotide polymorphisms; FH: familial hypercholesterolemia; P/LP: pathogenic/likely pathogenic; Probable FH: Dutch lipid clinic network (DLCN) score ≥6; Possible FH = DLCN score 3–5.

Participants for the proposed clinical trial will be drawn from the return of actionable variants empiric (RAVE) study in which 2537 adults (1791 with primary hypercholesterolemia, i.e., without a secondary cause such as hypothyroidism, hepatic disease, nephrotic syndrome, etc.) underwent sequencing of three genes that are implicated in FH: *LDLR*, *APOB*, and *PCSK9* [5] (Figure 1), in a central laboratory improvement amendment (CLIA)-certified laboratory. Participants found to have actionable variants after sequencing and variant identification is completed are notified of the results in a face to face meeting with a genetic counselor. A P/LP variant in one of the FH genes was identified in 24 individuals. An additional 26 FH patients who have detectable P/LP variants in the FH genes will be recruited from the Mayo FH Clinic or ongoing studies in which sequence data for *LDLR*, *APOB*, or *PCSK9* are available. From the RAVE study, we will enroll 50 patients with probable FH (a DLCN score ≥6) and 50 patients with possible FH (DLCN score 3–5) matched for age and sex. Participants will receive genetic results in a face-to-face meeting with a genetic counselor and discuss implications for family members. We will compare the number of first-degree relatives screened and new cases detected as well as costs of screening. New cases will be defined as first-degree relatives of index cases who have an LDL-C > 155 mg/dL (>130 mg/dL in children) or presence of a P/LP variant in one of the FH genes.

### 2.2. Participants

Using validated electronic phenotyping algorithms [6], we screened 51,652 Mayo Clinic BioBank participants and 10,000 participants from the Mayo Vascular Diseases Biorepository to identify individuals who met the following eligibility criteria: (1) residents of Southeast Minnesota; (2) LDL-C levels >155 mg/dL without an identifiable secondary cause of hypercholesterolemia; (3) age 18–78 years; (4) absence of severe comorbidity; (5) no major learning barriers, such as hearing impairment or dementia that would compromise their ability to give written informed consent. We identified 5010 patients who met these criteria and invited them to participate. We enrolled 2537 participants who underwent CLIA-certified targeted sequencing of 103 genes including three genes known to cause FH (*LDLR*, *APOB*, *PCSK9*) and genotyping of six single nucleotide polymorphisms (SNPs) associated with LDL-C. We identified 25 participants with definite FH (defined as presence of a P/LP variant in one of the three genes), 172 patients with probable FH (DLCN score ≥ 6) and 362 patients with possible FH (DLCN score of 3–5).

### 2.3. Case Definition

A DLCN score was calculated for each patient [2]. We will include 50 patients with definite FH patients (defined as presence of P/LP variant in *LDLR*, *APOB*, *PCSK9*). We will also include 50 patients with probable FH (DLCN score ≥ 6) and 50 patients with possible FH (DLCN score 3–5) who are (individually) age- and sex-matched with the definite FH individuals. The study coordinator will contact eligible patients informing them about the trial and inviting them to participate in the study. They will inform patients about the trial, confirm eligibility, and subsequently obtain written informed consent. The study coordinator will describe the format for disclosure of genetic test results.

### 2.4. Sequencing LDLR, APOB, and PCSK9 and Genotyping of Six SNPs Associated with Low Density Lipoprotein Cholesterol

DNA from RAVE study participants was sent to a CLIA-certified laboratory for targeted sequencing. The post-capture library DNA was subjected to sequence analysis on Illumina HiSeq platform (Illumina Inc., San Diego, CA, USA) for 100 bp paired-end reads. Quality control metrics of the sequencing data and the process for identifying P/LP variants (including structural variants) have been described elsewhere [5]. In FH^PV−^ individuals, we will calculate a polygenic score for LDL-C by estimating the combined effect of the six SNPs associated with LDL-C, as previously described [7]. The gene score uses the weighted sum of the risk allele (i.e., the LDL-C-raising allele), the weights used being the corresponding per-allele (risk) β coefficients reported in the literature. The score will be divided into quintiles and disclosed to the patient [5].

### 2.5. Disclosure of Genetic Test Results to Probands and Contacting Family Members

**Figure 2:**
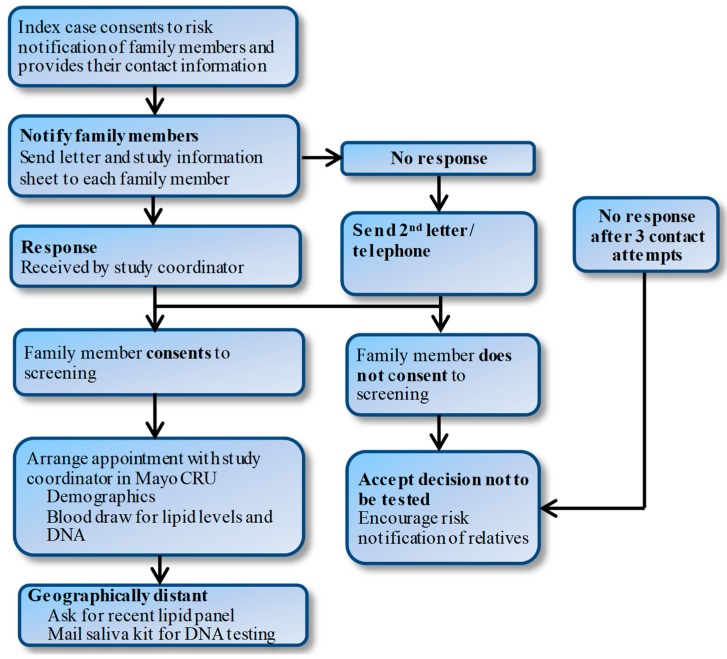
Proposed approach for cascade screening for FH. CRU: clinical research unit.

Study groups will receive the results of the genetic testing in a face-to-face meeting with a genetic counselor. The counselor will help participants to interpret and understand their results, highlighting the implications for family members. Patients will be consented to provide a list of first-degree family members and their contact information and permission to contact these family members by letter (Figure 2). The letter will state that one of their family members has been found to have elevated cholesterol levels and invite them to participate in the study. Individuals who do not respond will be contacted again two weeks later by letter and then a follow-up phone call if needed. For family members who are interested but geographically distant, we will schedule phone interviews.

### 2.6. Baseline Measures in Family Members

For family members who consent, we will obtain information about demographic factors, cardiovascular risk factors, such as diabetes, smoking, hyperlipidemia or obesity, family history, and medication use. A saliva kit (Color Genomics, Burlingame, CA, USA) will be used for DNA testing in family members of FH^PV+^ patients. Saliva test kits will be mailed to study participants who will then send the sample to Color Genomics. Once results are obtained, the family members will be notified. Family members of FH^PV−^ will be asked to have a lipid profile drawn, send results of a lipid profile drawn in the preceding 36 months, or permit access to their electronic health record (EHR) to obtain lipid levels.

### 2.7. Disclosure of Test Results to Family Members

**Figure 3:**
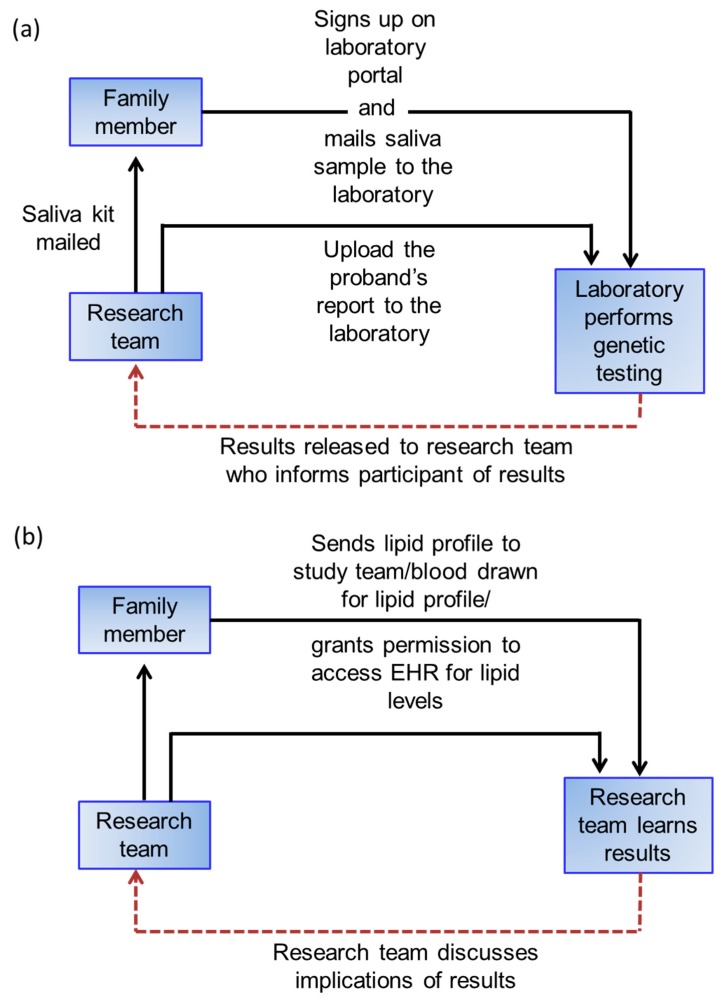
Approach for testing family members of patients with (**a**) definite FH; (**b**) probable/possible FH. EHR: electronic health record.

Family members that consent will receive results (DNA test results for definite FH families, lipid profile for probable/possible FH families) during a phone call by the study coordinator and will be encouraged to seek evaluation by their primary care provider (Figure 3). The study coordinator will be available as needed to help participants understand their results. Participants will also receive a letter that summarizes the results and the key points related to hypercholesterolemia as a risk factor for CHD.

### 2.8. Outcomes

The primary outcome will be the number of “new cases” detected in each group. New cases will be defined as LDL-C > 155 mg/dL in adults or >130 mg/dL in children in the probable/possible FH group and presence of P/LP variant in the definite FH group. For those family members already on a statin, LDL-C levels will be imputed as previously described [6]. A secondary outcome will be cost per new case detected. Analyses will be adjusted for age, sex, and LDL-C in the index case and size of the family. We will assess several relevant psychosocial measures using validated measures to enable comparison with existing literature [8]. Study staff assessing outcomes will be blinded to the FH status in the probands.

### 2.9. Cost Analyses

Decision tree analysis will be applied for comparison of cascade screening of the relatives of probands [9]. The primary outcome in the economic evaluation is the incremental cost-effectiveness ratio (ICER) in terms of US dollars per quality-adjusted life years (QALY) gained and per year of life saved for genetic screening compared with LDL-C facilitated screening [10]. We will assume that participants, including children >8 years old, with substantially elevated LDL-C would be started on statin treatment consistent with current guidelines [11]. The types of costs to be considered in the model include: screening, clinical consultation, genetic/clinical tests, treatments, follow-up visits, and disease costs, defined as cardiovascular events prevented based on LDL-C reduction. A Markov model with yearly cycles will be constructed to simulate the onset of first-ever CHD and death over a lifetime horizon. We will capture relatives’ post-study enrollment utilization of health care services by telephone interviews at 26 weeks and one year or by retrospective EHR review during the study period using observed utilization and beyond the study period using imputed values from the scientific literature. To maintain consistency and enhance the validity and interpretation of study findings, standardized costs will be assigned for each cardiovascular disease service/outcome based on Medicare allowable costs (i.e., assigned costs will include both the portion of the allowable costs reimbursed by the payer, as well as the portion that are the responsibility of the patient). Assigned costs will be those in effect at the time the analysis is conducted so as to be most relevant to patients, clinicians, and policy makers.

### 2.10. Statistical Methods

All survey data will be exported from REDCap (Research Electronic Data Capture) [12] to a SAS database for analyses. Data analysts will be blinded to the category of the FH participants. Descriptive data will be provided for all measures. The frequency (%) of categorical factors will be compared using either the chi-square or Fisher’s exact test. The yield of cascade screening will be expressed as the proportion of the index cases’ family members who have elevated lipids as defined above. This estimated proportion will be based on a weighted average of the proportions of each index case’s family members detected to have hypercholesterolemia or a P/LP variant. Weights will take into account intra-family correlation, using a random effects model via generalized linear models. If there is little “between-family” variation, then the weights will be nearly proportional to the number of family members. Conversely, if there is a great deal of “between-family” variation, then the weights will be more nearly equal. The random effects model will effectively result in weights on the family-specific proportions, that are somewhere between “equal” and “proportional to family size”.

### 2.11. Power

With 50 probands in each of the three groups (definite FH, probable FH, and possible FH), and if we assume an average of three family members per proband participating, we will assume an “effective” number of 1.5 family members per proband, based on the likelihood of intra-family correlation. This estimate is probably conservative. Thus, we estimate power based on 75 “independent” observations per group. We also assume an overall average yield of 20%. If we conduct a 3 × 2 chi-square test on the variation of yields across the three groups, and if the true yields are: 9.8% (possible FH), 20% (probable FH), and 30.2% (definite FH), then this configuration will yield 80% power to detect these differences. Of note, we are not assuming the groups associated with each rate, just the rates themselves. Another configuration with the same power would be: 14.1% (possible FH), 14.1% (probable FH), and 31.8% (definite FH).

## 3. Results

Using validated electronic phenotyping algorithms, we screened 29,352 Mayo Clinic BioBank participants to identify individuals who met the eligibility criteria described above. Participant characteristics are shown in Table 1. Compared to participants with probable FH, those with P/LP variants were younger.

## 4. Discussion

Familial hypercholesterolemia (FH) is relatively prevalent; in one study, the prevalence of possible FH (LDL-C > 190 mg/dL) was 7% [3], and in the Mayo Employee and Community Health cohort (*n* = 138,000), we found that 5% of the patients had LDL-C > 190 mg/dL [6]. FH is associated with an increased risk of CHD due to the marked elevation in LDL-C [13] and early detection and treatment of individuals with FH, who are often asymptomatic, is critical to prevent adverse outcomes such as sudden cardiac death and myocardial infarction. The ACC/AHA guideline recommends initiating statin therapy in patients with LDL-C > 190 mg/dL [11].

However, no guideline is available in the US for cascade screening of patients with FH including those with possible FH (LDL-C > 190 mg/dL). The European guideline recommends that all patients with clinical and biochemical features of FH should be counseled about DNA cascade screening in their relatives [4]. In the Netherlands, cascade testing in families of FH probands with a P/LP variant led to statin treatment of family members at risk of early CHD [14]. As a result >70% of the estimated familial cases have been identified in contrast to <5% in the US [15].

FH patients without an identifiable P/LP variant in *LDLR*, *APOB*, and *PCSK9* may have a polygenic etiology, although in some, a novel monogenic etiology may be operative [16]. In theory, the inclusion of index cases with a polygenic rather than monogenic cause of hypercholesterolemia would reduce the efficiency of a cascade screening program as the proportion of their relatives who are likely to also have raised LDL-C is less than the 50% predicted for monogenic FH. In one study [17], the proportion of relatives with elevated LDL-C was 30%, and further cascade testing from these individuals (e.g., their children) would be even less effective [18]. It has been hypothesized that cascade screening may be efficient in those with a low-polygenic score for LDL-C, and thereby more likely to have a monogenic etiology. Talmud et al. [16] proposed that cascade testing in FH patients should be restricted to those in the bottom 20% of the distribution since an LDL-C > 190 mg/dL is unlikely to be due to the inheritance of LDL-C-raising SNPs. However, no empiric data are available to support this statement. In fact, one can argue that if polygenic FH is itself heritable, then a higher polygenic score is more likely to yield family members with a higher polygenic score, which would lead to a higher yield of FH from cascade screening. Additional studies are needed to investigate whether a polygenic score for LDL-C is predictive of the yield of cascade screening.

The office of Public Health Genomics at the centers for Disease Control and Prevention has prioritized the detection of prevalent and actionable (i.e., tier 1) genetic disorders such as FH, but currently, no formal guidelines or recommendations in the United States exist regarding cascade testing for FH. Several factors that may impede cascade screening [19]. The use of genetic testing for FH in the US is very low, and patients and family members are concerned about the stigma associated with genetic diagnoses and potential implications for employment and health insurance coverage. Awareness of FH among health care providers is also low.

Because of the health insurance portability and accountability act (HIPAA), disclosing the risk of genetic disease to family members can incur liability, even if this knowledge leads to early detection and treatment. Direct contact of family members by health care providers with the proband’s consent [20] appears to be more effective than contact by the proband but is not implemented due to concern over violating HIPPA. For this study, we obtained IRB approval to contact family members after obtaining consent from the probands.

A limitation of our proposed clinical trial is that participants are primarily non-Hispanic whites. Additional studies will be needed in diverse ethnic groups. Due to logistical reasons, we are only able to perform genetic testing in family members of probands with a P/LP variant. However, plasma lipid levels will be obtained where feasible. To ascertain new cases, we proposed LDL-C cutoffs for children and adults, but we will also consider age and body mass index-dependency of LDL-C levels [21,22].

Clinical trials in genomics are critical to inform appropriate strategies to reduce the burden of common genetic disorders such as FH. We described the design of the CASH study, the first clinical trial to compare the yield of cascade screening in different categories of FH patients. The study, leveraging existing infrastructure of the ongoing RAVE study at Mayo [5], will provide new data regarding the yield and cost effectiveness of cascade screening for FH, a relatively prevalent condition that is associated with significantly increased risk of CHD.

## Figures and Tables

**Table 1:** Characteristics of patients with FH (*n* = 293).

	Definite FH *n* = 24	Probable FH *n* = 119	Possible FH *n* = 150
Age, y	45.2 ± 9.9	51.0 ± 8.3	49.9 ± 8.8
Age range, y	25.6–64.1	26.8–69.3	21.2–68.8
Women	13 (54.2%)	70 (58.9%)	84 (56.0%)
White	100%	118 (99.2%)	148 (98.7%)
LDL-C, mg/dL	237.4 ± 46.8	253.7 ± 16.3	211.1 ± 16.3
Statin use (baseline)	3 (12.5%)	38 (31.9%)	13 (8.7%)
Hypertension	14 (58.3%)	82 (68.9%)	81 (54.0%)
Type 2 diabetes	7 (29.2%)	37 (31.1%)	24 (16.0%)

Definite FH = presence of a pathogenic/likely pathogenic (P/LP) variant in one of the FH genes; Probable FH = DLCN score ≥6 without a P/LP variant; Possible FH = DLCN score 3–5, without a P/LP variant.
